# Effect of extended-release naltrexone on striatal dopamine transporter availability, depression and anhedonia in heroin-dependent patients

**DOI:** 10.1007/s00213-015-3891-4

**Published:** 2015-03-12

**Authors:** Eline R. Zaaijer, Lonneke van Dijk, Kora de Bruin, Anna E. Goudriaan, Laureen A. Lammers, Maarten W. J. Koeter, Wim van den Brink, Jan Booij

**Affiliations:** 1 Amsterdam Institute for Addiction Research, Department of Psychiatry, Academic Medical Center, University of Amsterdam, PO Box 22660, 1100 DD Amsterdam, The Netherlands; 2 Department of Nuclear Medicine, Academic Medical Center, University of Amsterdam, PO Box 22660, 1100 DD Amsterdam, The Netherlands; 3 Department of Hospital Pharmacy, Academic Medical Center, University of Amsterdam, PO Box 22660, 1100 DD Amsterdam, The Netherlands

**Keywords:** Dopamine transporter, Abstinence, Addiction, Brain imaging, In vivo, Opioid receptor

## Abstract

**Rationale:**

Extended-release naltrexone (XRNT), an opioid receptor antagonist, is successfully used in the treatment of opioid dependence. However, naltrexone treatment of opioid-dependent patients may reduce striatal dopamine transporter (DAT) availability and cause depression and anhedonia.

**Objectives:**

The aim of this study is to investigate changes in striatal DAT availability and symptoms of depression (Beck Depression Inventory (BDI)) and anhedonia (Snaith Hamilton Pleasure Scale (SHAPS)) before and during XRNT treatment.

**Methods:**

At baseline, ten detoxified heroin-dependent patients and 11 matched healthy controls underwent [^123^I]FP-CIT single photon emission computed tomography (SPECT) imaging to assess striatal DAT binding. Patients underwent a second SPECT scan 2 weeks after an intramuscular injection with XRNT.

**Results:**

At baseline, the mean binding potential (BP_ND_) in the putamen was at a trend level lower and the mean BDI score was significantly higher in heroin patients (*n* = 10) than in controls (*n* = 11) (3.45 ± 0.88 vs. 3.80 ± 0.61, *p* = 0.067, *d* = −0.48 and 12.75 ± 7.40 vs. 5.20 ± 4.83, *p* = 0.019, *d* = 1.24, respectively). Post hoc analyses in subgroups with negative urine analyses for opioids and cocaine showed significantly lower baseline putamen BP_ND_ in heroin patients (*n* = 8) than controls (*n* = 10) (3.19 ± 0.43 vs. 3.80 ± 0.64, *p* = 0.049, *d* = −1.03). XRNT treatment in heroin patients was not significantly associated with changes in striatal DAT availability (*p* = 0.348, *d* = 0.48), but the mean BDI score after XRNT treatment was significantly lower than before treatment (7.75 ± 7.21 vs. 12.75 ± 7.40, *p* = 0.004, *d* = −0.68).

**Conclusions:**

The results of this study suggest that XRNT treatment does not reduce striatal DAT availability and has no significant effect on anhedonia, but is associated with a significant reduction of depressive symptoms.

**Electronic supplementary material:**

The online version of this article (doi:10.1007/s00213-015-3891-4) contains supplementary material, which is available to authorized users.

## Introduction

The worldwide prevalence of opioid dependence is estimated to be 0.2 % (Degenhardt et al. 2014) and the prevalence of illicit opioid use 0.7 % (UNODC 2012). The main illicit opioid used in Europe is heroin. Although a downward trend in the use of heroin was suggested, existing problem users will remain a key issue for many years to come (EMCDDA 2013). In the Netherlands (16.7 million inhabitants), the estimated number of opioid-dependent people in 2012 was 14,000, which is approximately 1 per 1000 adult inhabitants (Cruts et al. 2013). More than 90 % of the opioid-dependent people in the Netherlands inhale heroin, and injection of heroin is rare (NDM 2012; Cruts et al. 2013).

In the Netherlands, about 80 % of all heroin-dependent people is in treatment, mostly methadone maintenance treatment (85 %) and heroin-assisted treatment (5 %) (Cruts et al. 2013; Wisselink et al. 2013). The remaining 10 % of patients in treatment are in some kind of abstinence-oriented program, including extended detoxification programs followed by outpatient psychosocial support and oral naltrexone (Cruts et al. 2013). Internationally, the focus of opioid addiction treatment is shifting toward recovery-oriented drug treatment (Neale et al. 2013), resulting in a greater emphasis on abstinence as the final treatment goal. However, outpatient treatment with or without oral naltrexone was associated with early treatment discontinuation and very high relapse rates. As a consequence, oral naltrexone was probably not more effective than placebo (Minozzi et al. 2011).

Extended-release naltrexone (XRNT), given as injection or implant, may be a more suitable treatment for opioid addiction than oral naltrexone treatment due to better compliance. XRNT implants and injections significantly reduced heroin use (Gastfriend 2011; Lobmaier et al. 2011) and opioid-dependent people receiving XRNT injections had significantly more opioid-free weeks than opioid-dependent patients who were given a placebo injection (Syed and Keating 2013). Patients receiving XRNT injections stayed in treatment longer than patients receiving placebo injections (Lobmaier et al. 2008; Syed and Keating 2013), and XRNT treatment was well tolerated (Krupitsky and Blokhina 2010; Gastfriend 2011).

Although naltrexone treatment compliance can be improved by extended-release formulations, there are concerns about possible side effects that may result in treatment dropout, i.e., no further injections/implants. For example, significantly higher 6β-naltrexol levels, the major metabolite of naltrexone, were found in subjects who experienced one or more side effects (i.e., headache, nausea, anxiety) (King et al. 1997). Moreover, the prevalence of depression and anhedonia was found to be high among heroin addicts (Tiurina et al. 2011). Endogenous opioids influence motivational and stress regulatory processes and mood regulation directly by binding to the μ-opioid receptor, which causes inhibition of the gamma-aminobutyric acid (GABA) neurons and indirectly induces dopamine release in the nucleus accumbens (Koob and Le Moal 2008). Naltrexone, which is a μ-opioid receptor antagonist, possibly disturbs normal endogenous opioid binding leading to reduced dopamine release. The dopamine transporter (DAT) plays an important role in controlling the synaptic dopamine levels by removing dopamine from the synapse. So, when the dopamine release is changed chronically, this may lead to changes in synaptic dopamine levels, and consequently to changes in the DAT expression (Schmitt and Reith 2010; Vaughan and Foster 2013). Interestingly, human studies also showed that high endogenous striatal DA release was associated with anhedonia (Zijlstra et al. 2008) and low availability of striatal DATs was associated with symptoms of apathy (David et al. 2008) and depression (Sarchiapone et al. 2006; Roselli et al. 2009). Additionally, opioid-dependent patients who were abstinent showed lower striatal dopamine D_2/3_ receptors (Zijlstra et al. 2008) and DAT availability compared to healthy controls, but it was not clear whether this effect was reversible (Jia et al. 2005; Shi et al. 2008; Yeh et al. 2012; Liu et al. 2013). Finally, there is evidence that chronic naltrexone administration in rats results in decrease of striatal DAT availability (Bhargava and Gudehithlu 1996). Therefore, (chronic) naltrexone treatment may further reduce striatal DAT availability leading to an exacerbation of existing depressive symptoms and anhedonia in opioid-dependent patients.

The effects of naltrexone on anhedonia in humans were mainly assessed with self-reports of pleasure ratings. These studies showed that oral naltrexone can cause anhedonia in healthy controls (Murphy et al. 1990; Daniel et al. 1992; Yeomans and Gray 2002). However, although XRNT treatment was associated with a reduction of the hedonic properties of addictive substances (O’Brien et al. 2010), XRNT treatment did not reduce the ability to experience pleasure during natural rewarding activities in addicted patients (O’Brien et al. 2010; Tiurina et al. 2011). In order to better understand these findings, we conducted the first study looking at the effect of XRNT on both striatal DAT binding and self-reported anhedonia in detoxified heroin addicts.

Based on the literature, we hypothesize that (1) at baseline, heroin-dependent patients have lower striatal DAT availability and report more anhedonia and depressive symptoms than healthy controls; (2) during XRNT treatment, heroin-dependent patients show a further decrease in striatal DAT availability compared to baseline and an increase in anhedonia and depression scores; (3) plasma levels of naltrexone and its major metabolite 6β-naltrexol in heroin-dependent patients correlate with changes in striatal DAT availability and with anhedonia/depression before and during XRNT treatment; (4) at baseline and at follow-up, striatal DAT availability is negatively correlated with anhedonia and depression; and (5) the decrease in striatal DAT availability during treatment is associated with an increase in anhedonia and depression.

## Methods

### Subjects

Subjects were recruited between January 2013 and July 2014. Twelve detoxified heroin-dependent patients (11 male) were recruited from addiction treatment centers throughout the Netherlands. Inclusion criteria were (1) diagnosis of DSM-IV opioid dependence, (2) heroin as the main substance of abuse, and (3) inhalation as the main route of administration of heroin. Exclusion criteria were (1) estimated IQ <70, (2) prior or current diagnosis of psychosis or current depression with suicidal ideation, (3) use of medication that interferes with binding of the DAT radiotracer, (4) use of naltrexone in the past 6 months, (5) history of head trauma or brain surgery, (6) (planned) pregnancy, breastfeeding, or no acceptable method of contraception, (7) involuntary treatment, (8) medical contradictions for XRNT, and (9) no intention to be opioid-free for a minimum of 10–14 days before starting XRNT treatment.

Eleven healthy subjects were included who had no diagnosis of substance dependence and were matched to the patient group for gender, age, body mass index (BMI), and smoking status. Healthy controls were recruited through online advertisement and flyer postings. Exclusion criteria for controls were identical to exclusion criteria for the heroin-dependent subjects.

All subjects provided written informed consent to participate in the study. The study was approved by the Ethical Committee of the Academic Medical Centre of the University of Amsterdam, where the study was conducted, and performed in accordance with the ethical standards laid down in the 1964 Declaration of Helsinki.

### Study design

For patients, there was a 2-week heroin- and methadone-free period between the end of detoxification and the first scanning day in order to minimize the risk of opioid withdrawal symptoms after XRNT injection. To measure DAT availability in vivo, the first [^123^I]FP-CIT single photon emission computed tomography (SPECT) was performed just before the XRNT injection and the second scan was made 2 weeks after the XRNT injection. In healthy control subjects, only one (baseline) SPECT scan was performed. All subjects were required to have a negative urine drug screen (UDS) for opioids, cocaine, and amphetamine on the day of the SPECT scan(s). None of the subjects used medication that could interfere with [^123^I]FP-CIT binding (Booij and Kemp 2008). A breath alcohol test was performed to assess acute alcohol intoxication.

### Clinical assessments

DSM-IV criteria for substance use disorders, psychotic disorders, and depressive disorder with suicidal ideation were assessed with the Dutch translation of the Mini-International Neuropsychiatric Interview (MINI; van Vliet and de Beurs 2007). Before each scan, subjects were asked to fill out self-report questionnaires assessing depressive symptoms (Beck Depression Inventory (BDI); Arnou et al. 2001) and anhedonia (Snaith-Hamilton Pleasure Scale (SHAPS); Snaith et al. 1995). On both questionnaires, a higher total score indicates more severe depression or anhedonia, respectively. Smoking status was assessed with the Fagerström Test for Nicotine Dependence (FTND; Heatherton et al. 1991). IQ was estimated with the Dutch Adult Reading test (Schmand et al. 1991).

### Study medication

After the first SPECT scan, patients were given an intramuscular injection with XRNT (Vivitrol®, Alkermes, Inc., USA). Extended-release naltrexone microspheres (Alkermes, Inc., USA) were administered as a 4-ml gluteal intramuscular injection containing 380 mg naltrexone. After injection, patients were kept at the research facility for 30 min to check whether they developed opioid withdrawal symptoms and to treat them if necessary. The second SPECT session was conducted 2 weeks after the XRNT injection. The timing of the session coincided with peak naltrexone levels (Krupitsky and Blokhina 2010). Plasma samples were taken on the day of the second SPECT session to assess peak naltrexone levels and its major metabolite 6β-naltrexol (Slawson et al. 2007).

### SPECT imaging procedure

SPECT brain imaging was performed on a brain-dedicated system. This system (Neurofocus) has 12 individual crystals equipped with a focusing collimator and a spatial resolution of approximately 6.5 mm full-width at half maximum throughout the 20-cm field of view. [^123^I]FP-CIT, which is a well-validated radiotracer for striatal DAT imaging (Booij et al. 1997), was injected intravenously at an approximate dose of 110 MBq. [^123^I] labeling and acquisition were performed as described previously (Tissingh et al. 1998). [^123^I]FP-CIT (GE Healthcare, Eindhoven, The Netherlands) had a specific activity of 750 MBq/nmol and a radiochemical purity >95 %. Image acquisition was performed 3 h after injection (Booij et al. 1999). Images were corrected for attenuation and reconstructed in 3D (de Win et al. 2005; Boot et al. 2008).

### Analysis of SPECT data

[^123^I]FP-CIT binding in the striatum was determined by analyzing the five consecutive transverse slices representing the most intense binding in the striatum. A standard region of interest (ROI) template (constructed according to a stereotactic atlas) including two regions representing DAT binding (caudate nucleus and putamen) and one region representing nonspecific binding (occipital cortex) was placed bilaterally on the images, as previously reported (de Win et al. 2005). Also, a standard template was used representing DAT binding in the striatum as a whole. Specific DAT versus nonspecific binding ratios (binding potential (BP_ND_); Innis et al. 2007) were calculated for caudate nucleus, putamen, and whole striatum using the following formula:

$$ {\mathrm{BP}}_{\mathrm{ND}}=\frac{\mathrm{mean}\left[{}^{123}\mathrm{I}\right]\mathrm{F}\mathrm{P}\hbox{-} \mathrm{C}\mathrm{I}\mathrm{T}\;\mathrm{binding}\;\mathrm{in}\;\mathrm{R}\mathrm{O}\mathrm{I}-\mathrm{mean}\left[{}^{123}\mathrm{I}\right]\mathrm{F}\mathrm{P}-\mathrm{C}\mathrm{I}\mathrm{T}\;\mathrm{binding}\;\mathrm{in}\;\mathrm{occipital}\;\mathrm{cortex}}{\mathrm{mean}\left[{}^{123}\mathrm{I}\right]\mathrm{F}\mathrm{P}\hbox{-} \mathrm{C}\mathrm{I}\mathrm{T}\;\mathrm{binding}\;\mathrm{in}\;\mathrm{occipital}\;\mathrm{cortex}} $$

### Statistical analysis

Normality of distribution of all data was tested with the Kolmogorov-Smirnov test. Equality of variances was tested with Levene’s test.

Group differences in baseline characteristics were assessed using an independent samples *t* test when a variable was normally distributed and a Mann-Whitney *U* test when a variable was not normally distributed. Correlations between BP_ND_ of the left and right striatum was assessed using Pearson’s and Spearman’s correlation coefficients.

BP_ND_ in ROIs and BDI and SHAPS scores were compared between groups using an independent samples *t* test when a variable was normally distributed and a Mann-Whitney *U* test when a variable was not normally distributed.

Within the patient group, analyses were performed using a paired samples *t* test. Within the patient group, we calculated Pearson’s *r* between naltrexone and 6β-naltrexol plasma levels and change in BP_ND_ (in striatum, caudate, and putamen), BDI, and SHAPS scores between scans. Also, Pearson’s *r* were calculated between BP_ND_ (in striatum, caudate, and putamen) and BDI scores/SHAPS scores.

We reported effect sizes (*d* values; Cohen 1977) for each test because of the relatively small sample size, where *d* = 0.2 is considered a small effect, *d* = 0.5 a medium effect, and *d* = 0.8 a large effect.

All statistical analyses were performed using IBM Statistical Package for the Social Sciences (SPSS) version 20, and statistical significance was defined as *p* < 0.05. Given the small number of subjects, correction for multiple testing was not performed to prevent increased type II errors, resulting in low power.

## Results

### Urine drug screen

Five subjects tested positive for drugs: two heroin-dependent patient tested positive for cocaine and opioids on both scanning days, two heroin-dependent patients tested positive for opioids on the first scanning day and one healthy control tested positive for opioids. The healthy control, who tested positive for opioids, indicated that he had used codeine the day before scanning. Since cocaine interferes with [^123^I]FP-CIT binding (Booij and Kemp 2008), the two patients testing positive for cocaine were excluded from further analyses. Opioid use could possibly influence DAT availability (Booij and Kemp 2008). Therefore, analyses were performed twice: (1) in the first analysis, the two patients testing positive for cocaine were excluded, resulting in 10 patients and 11 controls; (2) in the second analysis, also all subjects testing positive for opioids were left out, resulting in 8 patients and 10 controls. Missing urinalysis data were imputed as positive. As a consequence, one more patient with missing urinalysis data on the day of the second scan was excluded from some of the analyses, resulting in seven patients for the within-patient comparison. All subjects had a negative alcohol breath test on the scan day(s).

### Sample characteristics

Demographic and clinical characteristics are displayed in Table 1. In the first analysis, no significant differences were found in gender, age, BMI, and smoking status (FTND) between the patients and healthy controls. After excluding patients testing positive for cocaine and opioids, differences between groups for all characteristics even decreased, except for FTND. Since smoking status may influence striatal BP_ND_ (Danielson et al. 2011), we corrected for FTND in the between-group analyses with SPECT data.

**Table 1:** Demographic and clinical characteristics of healthy controls and heroin patients

Demographics and clinical characteristics of cocaine-free subjects (defined as negative for cocaine on urine analysis)					
	Healthy controls (*n* = 11)	Heroin patients (*n* = 10)^a^	*t* (*df* = 19)/U	*p* value	Cohen’s *d*
Sex (nr male)	11	10			
Age (mean ± SD) (years)	45.6 ± 9.4 (range 29–56)	44.9 ± 5.5 (range 37–53)	−0.216	0.832	−0.09
Duration of heroin dependence (mean ± SD) (years)	N/A	16.6 ± 8.8 (range 2–30)			
Body mass index (BMI) (mean ± SD) (kg/m^2^)	26.4 ± 4.5	24.5 ± 4.4	37.000	0.202	−0.42
Fagerström Test for Nicotine Dependence (FTND)	0.73 ± 0.65	0.60 ± 0.70	48.500	0.612	−0.19
Demographics and clinical characteristics of opioid-free subjects (defined as negative for both cocaine and opioids on urine analysis)					
	Healthy controls (*n* = 10)	Heroin patients (*n* = 8)	*t* (*df* = 16)/U	*p* value	Cohen’s *d*
Sex (nr male)	10	8			
Age (mean ± SD) (years)	45.2 ± 9.8 (range 29–56)	45.1 ± 6.0 (range 37–53)	−0.019	0.985	−0.01
Duration of heroin dependence (mean ± SD) (years)	N/A	17.0 ± 9.5 (range 2–30)			
Body Mass Index (BMI) (mean ± SD) (kg/m^2^)	25.7 ± 4.2	24.3 ± 4.0	29.000	0.325	−0.35
Fagerström Test for Nicotine Dependence (FTND)	0.80 ± 0.63	0.63 ± 0.74	33.500	0.523	−0.25

Cohen’s *d*: 0.20 = small, 0.50 = moderate, 0.80 = large (Cohen 1977)

*N/A* not applicable

^a^Excluding two patients that tested positive on cocaine use at the time of the scan

### Binding potential (BP_ND_) in regions of interests

**Fig. 1:**
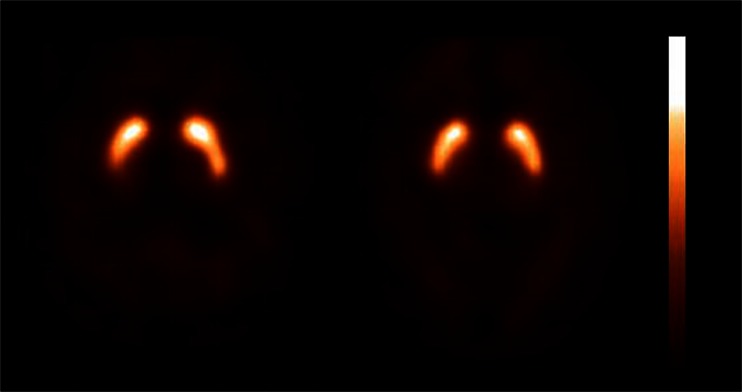
[^123^I]FP-CIT SPECT images (transversal slides at the level of the striatum) of a typical heroin-dependent patient, before (*left image*) and 2 weeks after (*right image*) an intramuscular injection with XRNT (380 mg). Note that visual analyses of the images did not show differences between the two conditions, which was confirmed by the quantitative analyses (see “Results” section)

In all participants, intense [^123^I]FP-CIT binding was observed in the striatum bilaterally (Fig. 1). DAT availability in the left and right caudate nucleus/putamen/total striatum were highly correlated (*r* > 0.85, *p* < 0.05); therefore, the mean BP_ND_ of the bilateral measures was calculated and used in all analyses.

In the first analysis (all subjects except the two patients with a positive urine for cocaine), two patients did not have a second SPECT scan: one patient withdrew consent and quit the study before the second SPECT scan, the other patient had a missing urine sample on the day of the second SPECT scan, resulting in eight patients for the within-patient comparison. In the second analysis (all subjects except those with a positive urine for cocaine or opioids), only one of the remaining patients did not have a second SPECT scan and was therefore not included in within-patient comparison of BP_ND_ in ROIs before and during XRNT treatment (Table 2).

**Table 2:** BP_ND_ per ROI for controls and heroin patients (mean ± SD)

BP_ND_ (mean ± SD) for cocaine-free subjects (defined as negative for cocaine on urine analysis)			*p* value	Cohen’s *d*
PB vs HC	PB (*n* = 10)	HC (*n* = 11)		
Striatum, whole	3.64 ± 1.00	3.82 ± 0.63	0.139	−0.21
Caudate nucleus	3.62 ± 0.72	3.97 ± 0.85	0.321	−0.44
Putamen	3.45 ± 0.88	3.80 ± 0.61	0.067	−0.48
PO vs PB	PO (*n* = 8)	PB (*n* = 8)		
Striatum, whole	3.60 ± 0.59	3.65 ± 1.12	0.901	−0.05
Caudate nucleus	3.60 ± 0.62	3.62 ± 0.81	0.965	−0.02
Putamen	3.42 ± 0.72	3.42 ± 0.99	0.999	0.00
BP_ND_ (mean ± SD) for opioid-free subjects (defined as negative for both cocaine and opioids on urine analysis)			*p* value	Cohen’s *d*
PB vs HC	PB (*n* = 8)	HC (*n* = 10)		
Striatum, whole	3.36 ± 0.47	3.82 ± 0.66	0.155^a^	−0.72^a^
Caudate nucleus	3.45 ± 0.52	3.99 ± 0.90	0.198^a^	−0.63^a^
Putamen	3.19 ± 0.43	3.80 ± 0.64	0.049^a^	−1.03^a^
PO vs PB	PO (*n* = 7)	PB (*n* = 7)		
Striatum, whole	3.53 ± 0.60	3.28 ± 0.44	0.348	0.48
Caudate nucleus	3.55 ± 0.65	3.39 ± 0.53	0.579	0.27
Putamen	3.28 ± 0.65	3.10 ± 0.38	0.477	0.35

Cohen’s *d*: 0.20 = small, 0.50 = moderate, 0.80 = large (Cohen 1977). Nonparametric test for PB vs HC: striatum and putamen in the cocaine-free subjects’ analyses. Parametric tests were used for all other analyses listed. Means represent observed data that were not adjusted for FTND

*PB* patients at baseline, *HC* healthy controls, *PO* patients on XRNT treatment

^a^Adjusted for FTND scores

In the first analysis (including opioid positive subjects), we did not find significant group differences in baseline BP_ND_. However, in the second analysis (excluding both cocaine and opioid positive subjects), baseline BP_ND_ in the putamen of heroin patients was significantly lower than in healthy controls (*t*(16) = −2.301, *p* = 0.049, *d* = −1.03). There were no significant differences in BP_ND_ between healthy controls and heroin patients at baseline for other ROIs. Correction for FTND slightly decreased the difference in baseline BP_ND_ between heroin-dependent patients and healthy controls. Therefore, adjusted *p* values and effect sizes are displayed in Table 2.

We found no significant differences in BP_ND_ between heroin patients at baseline and after 2 weeks of XRNT treatment (Fig. 1), neither when opioid-positive patients were included nor when opioid-positive patients were excluded from the analysis (Table 2).

Since our main group of interest is heroin-dependent patients that are abstinent during the study, further analyses (see below) were only conducted for cocaine and opioid-free subjects.

### Depression and anhedonia

At baseline, BDI scores were significantly higher for heroin-dependent patients than for healthy controls (*t*(16) = 2.614, *p* = 0.019, *d* = 1.24), see Table 3. For heroin-dependent patients after 2 weeks of XRNT treatment, BDI scores were significantly lower than before treatment (*t*(7) = 4.132, *p* = 0.004, *d* = −0.68). There was no significant difference between groups for baseline SHAPS scores. Also, there were no significant differences between SHAPS scores before and after 2 weeks of XRNT treatment (Table 3).

**Table 3:** Beck Depression Inventory scores and Snaith-Hamilton Pleasure Scale scores for healthy controls and heroin patients (mean ± SD) that had a negative UDS for cocaine and opioids

	HC (*n* = 10)	PB ( *n* = 8)	PO (*n* = 8)	*p* value (Cohen’s *d*)	
				PB vs HC	PO vs PB
BDI	5.20 ± 4.83	12.75 ± 7.40	7.75 ± 7.21	0.019 (1.24)	0.004 (−0.68)
SHAPS	24.00 ± 5.74	24.88 ± 5.22	22.75 ± 6.71	0.742 (0.16)	0.326 (−0.35)

Cohen’s *d*: 0.20 = small, 0.50 = moderate, 0.80 = large (Cohen 1977)

*PB* patients at baseline, *HC* healthy controls, *PO* patients on XRNT treatment, *BDI* Beck Depression Inventory, *SHAPS* Snaith-Hamilton Pleasure Scale

### Naltrexone and 6β-naltrexol plasma levels

Plasma data were missing for one patient due to technical reasons. No significant correlations were found between naltrexone/6β-naltrexol plasma levels and change in BP_ND_ (in whole striatum, caudate nucleus, and putamen), BDI and SHAPS scores in heroin-dependent patients between scans (supplementary data, Tables S1 and S2).

### Integration of SPECT data and behavioral parameters

No significant correlations were found for striatal DAT binding and anhedonia or depression at baseline or at follow-up. Correlations between decrease in striatal DAT binding during treatment and increase in anhedonia and depression were not calculated because we did not find a decrease in striatal DAT binding nor an increase in anhedonia and depression.

## Discussion

The current study is the first to assess the effects of XRNT treatment on striatal DAT binding and self-reported depression and anhedonia in heroin-dependent subjects. Our present results suggest that blocking of the μ-opioid receptor by XRNT does not decrease striatal DAT binding and does not increase self-reported anhedonia, but is associated with a significant decrease in depressive symptoms.

In line with our first hypothesis, we found significantly lower DAT binding at baseline in the putamen of detoxified heroin-dependent patients with a negative urine test for opioids compared to controls, which is in line with previous studies (Jia et al. 2005; Shi et al. 2008; Yeh et al. 2012; Liu et al. 2013, Table 4). This implicates that detoxified heroin patients have lower striatal DAT availability. This reduction in DAT availability compared to controls may be related to long-term heroin abuse since patients and healthy controls were matched for other variables influencing DAT availability. However, due to the design of our study, we cannot exclude the possibility of preexisting differences in DAT availability. Although the lower DAT binding in heroin-dependent patients was not significant for the caudate nucleus and whole striatum, effect sizes indicate moderate to large effects of long-term heroin abuse on DAT binding, supporting the hypothesis that differences in these areas may be found when larger sample sizes are included (Table 4; Liu et al. 2013).

**Table 4:** Overview of literature about DAT imaging in opioid dependent patients

Author	Journal	Number	Tracer	DAT availability (*p*)	Abstinent before and during study? (yes/no)
Liu et al. (2013)	Psychopharmacology	64 heroin-dependent patients (43 completed study) 15 healthy controls	[^99m^Tc]TRODAT-1 (SPECT)	LC RC LP RP Control vs heroin patients <0.001 < 0.001 < 0.001 < 0.001	Yes, 15–18 days of detoxification before start of study Abstinence was confirmed by urine and blood screens
Shi et al. (2008)	Eur. Journal of Pharmacology	11 heroin-detoxified subjects that were in prolonged abstinence (PA) 10 heroin-dependent subjects in methadone maintenance treatment (MMT) 10 healthy controls (HC)	[^11^C]CFT (PET)	LC RC LP RP HC vs MMT 0.040 0.034 0.011 0.044 HC vs PA 0.008 0.009 0.151 0.533 MMT vs PA 0.800 0.911 0.032 0.024	Yes, >6 months heroin-free before scanning Abstinence was recorded by urine screening at start of study
Yeh et al. (2012)	Psychopharmacology	16 low-dose methadone users 12 methadone-free abstainers 32 healthy controls	[^99m^Tc]TRODAT-1 (SPECT)	Striatum Control vs meth-free 0.048 Control vs low-dose meth <0.001 Low-dose meth vs meth-free 0.39	Unknown
Jia et al. (2005)	Addiction Biology 2005	36 heroin-dependent subjects 21 healthy controls	[^99m^Tc]TRODAT-1 (SPECT)	Ra^a^ Control vs Patient at baseline <0.05 Control vs Patient + Chinese herb >0.05 Patient baseline vs Chinese herb <0.01	Yes, >10 days of detox before start of study Abstinence was confirmed with urine morphine tests
Cosgrove et al. (2010)	Psychiatric Research: Neuroimaging	8 heroin-dependent subjects 8 healthy controls	[^123^I]β-CIT (SPECT)	Striatum Control vs heroin sub 0.20	No. Heroin positive at intake, last use of heroin before SPECT scan was not recorded
Kish et al. (2001)	Neuropharmacology	9 chronic heroin users 14 controls Postmortem striata	[^3^H]WIN 35,428	B_max_ of [^3^H] WIN 35,428 Caudate 0.41 Putamen 0.50	No. Presence of heroin metabolites in autopsied material
Liang et al. (2014)	Addiction Biology	20 heroin-dependent subjects 20 healthy controls	[^99m^Tc]TRODAT-1 (SPECT)	LC RC LP RP Control vs heroin patients <0.001 < 0.001 0.676 < 0.001	No. Used heroin within 24 h before SPECT scans, confirmed with urine tests

*LC* left caudate nucleus, *RC* right caudate nucleus, *LP* left putamen, *RP* right putamen, *SPECT* single-photon emission computed tomography, *PET* positron emission tomography

^a^Ra is the ratio of corpus striatum/the whole brain = striatal binding/whole brain binding

One healthy control indicated that he had used paracetamol with codeine for pain relief only on the day before scanning (two to three tablets). Exclusion of this subject from the analysis did not change mean striatal DAT binding of healthy controls. However, when we excluded the two heroin-dependent subjects who had a positive urine test for opioids, mean striatal DAT binding decreased and variation in DAT binding (SD) halved, indicating that acute use of codeine may not influence striatal DAT binding, while acute opioid use (i.e., heroin) may have a significant influence on striatal DAT binding. Indeed, acute opioid use increased striatal DA release (Di Chiara and Imperato 1988; Wise et al. 1995) and consequently may influence striatal DAT expression. One of the reasons that acute administration of the opioid receptor agonist codeine may not influence striatal DAT binding, while other opioids may do, might be that the affinity of codeine to the μ-opioid receptor is simply too low (*K*_*i*_ approximately 79 nmol/l; Raynor et al. 1993) to induce indirect changes in DAT expression. In contrast, although heroin itself has a low affinity for the μ-opioid receptor, once in the brain, it is hydroxylated to morphine (Yu 1996). Morphine has a high affinity for the μ-receptor (*K*_*i*_ approximately 14 nmol/l; Raynor et al. 1993) and might consequently indirectly influence DAT expression. Indeed, acute or subchronic treatment with another high-affinity μ-opioid agonist, namely fentanyl (*K*_*i*_ approximately 0.39 nmol/l; Raynor et al. 1993), decreased in vivo striatal DAT binding (Bergstrom et al. 1998). Thus, our present data may indicate that it is relevant to analyze a homogeneous group of subjects who are all truly and fully abstinent for opioids if one is interested to study DAT availability.

In Table 4, we summarized the findings of DAT imaging studies in heroin-dependent patients.

Our results from the analyses excluding subjects with a positive urine test for opioids (i.e., lower DAT binding in the putamen in the heroin-dependent patients) are in line with previous studies showing lower striatal DAT binding in abstinent heroin-dependent patients compared to healthy controls (Table 4: Jia et al. 2005; Shi et al. 2008; Yeh et al. 2012; Liu et al. 2013). In contrast, when subjects with a positive urine test for opioids were included in the analyses, our results are more consistent with the results from Cosgrove et al. (2010) who included heroin-dependent people testing positive for heroin (Table 4). Also, Kish and coworkers did not show lower DAT binding in a postmortem study in which eight out of the nine subjects died due to a heroin intoxication (Kish et al. 2001), although this is not in line with the results of a recent SPECT study (Liang et al. 2014). This again may stress the potential effect of current use of opioids on striatal DAT availability, as discussed earlier. Our findings underscore the fact that homogeneity of drug use/abstinence in the heroin-dependent subjects is needed for a correct interpretation of results in this field of research.

Opioids inhibit the release of dopamine, serotonin, acetylcholine, and norepinephrine, neurotransmitters that all may play an important role in the pathophysiology of depression (Miotto et al. 2002), and there is a high prevalence of depression and anhedonia in heroin-dependent patients (Tiurina et al. 2011). In line with these studies and our first hypothesis, we found higher levels of self-reported symptoms of depression in heroin-dependent subjects before XRNT treatment compared to healthy controls. However, in contrast to our expectation, no significant differences were found in anhedonic symptoms between the heroin-dependent patients and healthy controls at baseline. This might be explained by the fact that our healthy controls had a higher mean score for SHAPS than was previously reported for healthy controls (Franken et al. 2007). Another explanation may be that long-term opioid use increases (certain) depressive symptoms but not anhedonia.

Our second hypothesis, that during XRNT treatment, heroin-dependent patients will show a decrease in striatal DAT binding compared to baseline and that this decrease is associated with an increase in anhedonia and depression, was not confirmed. This hypothesis was based on findings of a previous in vitro rodent study (Bhargava and Gudehithlu 1996) in which a decreased striatal DAT availability after XRNT treatment was reported using the DAT ligand [^3^H]GBR 12935. Although this is the first study conducted with [^123^I]FP-CIT SPECT to image DAT binding during XRNT treatment in humans, results implicate that XRNT treatment does not decrease DAT availability. This is consistent with a recent rodent study (Zaaijer et al. 2015), in which rats were treated with short acting naltrexone or vehicle for 10 days, and no significant difference between groups was found in striatal DAT availability using [^123^I]FP-CIT storage phosphor imaging. However, we cannot rule out influences of XRNT on other parts of the dopaminergic system, e.g., on dopamine receptor availability. Importantly, although studies demonstrated that naltrexone induced anhedonia and depressive symptoms in healthy volunteers (Hollister et al. 1981; Murphy et al. 1990; Daniel et al. 1992; Yeomans and Gray 2002), our study and other studies investigating the influence of XRNT treatment on anhedonia in heroin-dependent people did not find a significant increase in anhedonia during XRNT treatment (O’Brien et al. 2010; Tiurina et al. 2011). In our study, depressive symptoms improved significantly after XRNT treatment. This is in line with results from Dean et al. (2006) and Mysels et al. (2011) who reported a decrease in depressive symptoms in heroin-dependent subjects that adhered to naltrexone treatment compared to baseline depressive symptoms (Dean et al. 2006; Mysels et al. 2011). This can either mean that XRNT treatment improves depressive symptoms or simply that abstinence from illicit opioid use improves depressive symptoms caused by long-term illicit opioid use, or that other factors are involved as well such as improvement of personal life circumstances. Finally, it cannot be excluded that the reduction in depressive symptoms is a result of positive expectations or normal fluctuations. In order to clarify this issue, a randomized placebo controlled trial is needed, although we understand the ethical issues involved in such an experiment. However, whatever the reasons may be, the current study supports previous findings that treatment with extended-release naltrexone does not lead to or worsens depressive symptoms.

In contrast to our third hypothesis, we found no significant correlations between naltrexone/6β-naltrexol plasma levels, striatal DAT binding, and BDI/SHAPS scores. In addition, contradictory to our fourth and fifth hypotheses, there was no significant relation between striatal DAT binding and anhedonia or depression at baseline for healthy controls and heroin-dependent subjects, and no relation between changes in DAT binding and changes in depression and anhedonia for heroin-dependent patients during XRNT treatment.

The main limitation of this study is the small sample size. However, after excluding subjects with a positive urine test for opioids, we found moderate to large effect sizes in the between-group analyses, indicating that significant differences would have been found with a larger sample size (Table 2). Another important limitation is that this study did not include a placebo arm to control for expectations and normal fluctuations in DAT SPECT and behavioral parameters. However, given the treatment opportunities that are currently available, such a strategy raises serious medical ethical issues. Further limitations include the following: (1) the absence of coregistration of SPECT images with MRI—coregistration of SPECT images with MRI may have improved the accuracy of placement of the ROIs; (2) the use of only one particular dose of XRNT and thus no possibility to study dose-effect relationships; (3) the use of only a single injection of XRNT to study changes in striatal DAT binding after 2 weeks of treatment; (4) no female subjects—sex-dependent effects on striatal DAT availability could not be accounted for; (5) no information on changes in personal circumstances related to increased/decreased anhedonic and depressive symptoms; and (6) restriction to inhalation as the route of heroin administration, while worldwide injection is preferred over inhalation and it could be that our results are not representative for heroin-dependent patients who inject heroin.

In conclusion, our results suggest that XRNT treatment in detoxified heroin-dependent patients does not decrease striatal DAT or increase anhedonia significantly, but is associated with a significant reduction of depressive symptoms.

## Electronic supplementary material

Below is the link to the electronic supplementary material.

ESM 1 (DOC 40 kb)
