# Supplementary material for: Effect of extended-release naltrexone on striatal dopamine transporter availability, depression and anhedonia in heroin-dependent patients
Source: Psychopharmacology (Berl). 2015 Mar 12;232(14):2597–607. doi: 10.1007/s00213-015-3891-4 (PMC4480848; doi:10.1007/s00213-015-3891-4)
Supplement: Supplementary file 1 — (DOC 40 kb) [file 213_2015_3891_MOESM1_ESM.doc]

Title: Effect of extended-release naltrexone on striatal dopamine transporter availability, depression and anhedonia in heroin dependent patients

List of authors:

Eline R Zaaijer^1, 2^, Lonneke van Dijk^1^, Kora de Bruin^2^, Anna E Goudriaan^1^, Laureen A Lammers^3^
Maarten WJ Koeter^1^, Wim van den Brink^1^ , Jan Booij^2^

Author affiliations and addresses:

^1^ Amsterdam Institute for Addiction Research, Department of Psychiatry, Academic Medical Center, University of Amsterdam, PO Box 22660, 1100 DD Amsterdam, The Netherlands

^2^ Department of Nuclear Medicine, Academic Medical Center, University of Amsterdam, PO Box 22660, 1100 DD Amsterdam, The Netherlands

^3^ Department of Hospital Pharmacy, Academic Medical Center, University of Amsterdam, PO Box 22660, 1100 DD Amsterdam, The Netherlands

Corresponding author:

Eline R. Zaaijer, MD
Departments of Psychiatry and Nuclear Medicine
Room F2-233
Academic Medical Center, University of Amsterdam
Meibergdreef 9
1105 AZ Amsterdam
The Netherlands
Tel. +31-20-5668322
ezaaijer@gmail.com

**Table S1** Correlations between naltrexone/6β-naltrexol plasma levels and change in BP_ND_ (in whole striatum, caudate nucleus and putamen; expressed as means of bilateral sides) in heroin dependent patients (n=6) between scans.

| naltrexone | | | 6β-naltrexol | |
| --- | --- | --- | --- | --- |
|  | r | *P* value | r | *P* value |
| Striatum, whole | 0.269 | 0.607 | 0.016 | 0.975 |
| Caudate nucleus | 0.339 | 0.511 | -0.064 | 0.904 |
| Putamen | 0.094 | 0.855 | -0.137 | 0.796 |

**Table S2 Correlations between naltrexone/6β-naltrexol plasma levels and change in BDI and SHAPS scores in heroin dependent patients (n=8) between scans.**

| naltrexone | | | 6β-naltrexol | |
| --- | --- | --- | --- | --- |
|  | r | *P* value | r | *P* value |
| BDI | -0.450 | 0.263 | -0.141 | 0.738 |
| SHAPS | 0.214 | 0.611 | 0.070 | 0.868 |

BDI= Beck depression Inventory, SHAPS= Snaith-Hamilton Pleasure Scale
